# HCCDB v2.0: Decompose Expression Variations by Single-cell RNA-seq and Spatial Transcriptomics in HCC

**DOI:** 10.1093/gpbjnl/qzae011

**Published:** 2024-02-03

**Authors:** Ziming Jiang, Yanhong Wu, Yuxin Miao, Kaige Deng, Fan Yang, Shuhuan Xu, Yupeng Wang, Renke You, Lei Zhang, Yuhan Fan, Wenbo Guo, Qiuyu Lian, Lei Chen, Xuegong Zhang, Yongchang Zheng, Jin Gu

**Affiliations:** Eight-Year Program of Clinical Medicine, Peking Union Medical College Hospital, Chinese Academy of Medical Sciences & Peking Union Medical College, Beijing 100006, China; MOE Key Laboratory of Bioinformatics, BNRIST Bioinformatics Division, Institute for Precision Medicine & Department of Automation, Tsinghua University, Beijing 100084, China; MOE Key Laboratory of Bioinformatics, BNRIST Bioinformatics Division, Institute for Precision Medicine & Department of Automation, Tsinghua University, Beijing 100084, China; Department of Liver Surgery, Peking Union Medical College Hospital, Chinese Academy of Medical Sciences & Peking Union Medical College, Beijing 100730, China; Department of Liver Surgery, Peking Union Medical College Hospital, Chinese Academy of Medical Sciences & Peking Union Medical College, Beijing 100730, China; Fuzhou Institute for Data Technology, Fuzhou 350207, China; Fuzhou Institute for Data Technology, Fuzhou 350207, China; Fuzhou Institute for Data Technology, Fuzhou 350207, China; Fuzhou Institute for Data Technology, Fuzhou 350207, China; MOE Key Laboratory of Bioinformatics, BNRIST Bioinformatics Division, Institute for Precision Medicine & Department of Automation, Tsinghua University, Beijing 100084, China; MOE Key Laboratory of Bioinformatics, BNRIST Bioinformatics Division, Institute for Precision Medicine & Department of Automation, Tsinghua University, Beijing 100084, China; University of Michigan – Shanghai Jiao Tong University Joint Institute, Shanghai Jiao Tong University, Shanghai 200240, China; Department of Automation, Shanghai Jiao Tong University, Shanghai 200240, China; International Cooperation Laboratory on Signal Transduction, Eastern Hepatobiliary Surgery Institute, Second Military Medical University, Shanghai 200438, China; National Center for Liver Cancer, Shanghai 201805, China; MOE Key Laboratory of Bioinformatics, BNRIST Bioinformatics Division, Institute for Precision Medicine & Department of Automation, Tsinghua University, Beijing 100084, China; Department of Liver Surgery, Peking Union Medical College Hospital, Chinese Academy of Medical Sciences & Peking Union Medical College, Beijing 100730, China; MOE Key Laboratory of Bioinformatics, BNRIST Bioinformatics Division, Institute for Precision Medicine & Department of Automation, Tsinghua University, Beijing 100084, China

**Keywords:** Hepatocellular carcinoma, Database, Integrative analysis, Single-cell RNA sequencing, Spatial transcriptomics

## Abstract

Large-scale transcriptomic data are crucial for understanding the molecular features of hepatocellular carcinoma (HCC). Integrated 15 transcriptomic datasets of HCC clinical samples, the first version of HCC database (HCCDB v1.0) was released in 2018. Through the meta-analysis of differentially expressed genes and prognosis-related genes across multiple datasets, it provides a systematic view of the altered biological processes and the inter-patient heterogeneities of HCC with high reproducibility and robustness. With four years having passed, the database now needs integration of recently published datasets. Furthermore, the latest single-cell and spatial transcriptomics have provided a great opportunity to decipher complex gene expression variations at the cellular level with spatial architecture. Here, we present HCCDB v2.0, an updated version that combines bulk, single-cell, and spatial transcriptomic data of HCC clinical samples. It dramatically expands the bulk sample size by adding 1656 new samples from 11 datasets to the existing 3917 samples, thereby enhancing the reliability of transcriptomic meta-analysis. A total of 182,832 cells and 69,352 spatial spots are added to the single-cell and spatial transcriptomics sections, respectively. A novel single-cell level and 2-dimension (sc-2D) metric is proposed as well to summarize cell type-specific and dysregulated gene expression patterns. Results are all graphically visualized in our online portal, allowing users to easily retrieve data through a user-friendly interface and navigate between different views. With extensive clinical phenotypes and transcriptomic data in the database, we show two applications for identifying prognosis-associated cells and tumor microenvironment. HCCDB v2.0 is available at http://lifeome.net/database/hccdb2.

## Introduction

Hepatocellular carcinoma (HCC), which accounts for the vast majority (75%–85%) of primary liver cancer, is one of the leading digestive system malignancies [1]. The accumulation of transcriptomic data in HCC has facilitated the precise subtyping and biomarker identification [2–4]. However, due to the high heterogeneity of HCC, transcriptomic data from a single cohort frequently generate inconsistent results due to limited sample size. The meta-analysis is an important approach for identifying stable patterns and cohort-specific effects across different datasets [5]. To provide a resource for studying the heterogeneities and dysregulated biological processes in HCC, we developed HCCDB v1.0, which integrated transcriptomes of 3917 samples from 15 bulk datasets and emphasized the centrality of meta-analysis in transcriptomic analysis [6–8]. With the growth of published datasets of HCC clinical samples over the past four years, it is imperative to increase the volume of the database contents. Moreover, it is interesting to assess the stability or reproducibility of the meta-analysis results after adding new datasets.

The bulk transcriptomic data provide important resources for analyzing gene expression variations of tumors in terms of malignancy, aggressiveness, and cell composition. However, bulk data only provide the average gene expression levels of the sample, which consists of multiple cell types and tumor subclones. The latest single-cell RNA sequencing (scRNA-seq) and spatial transcriptomics (ST) can decompose gene expression variations at the cellular level and obtain the spatial distribution of intact tissues. The scRNA-seq technique captures the cellular heterogeneity within the same tissue and reveals distinct cell subpopulations [9,10]. ST preserves the spatial location information of tumor tissues by *in situ* characterization of tissue spots, shedding light on integrating the functional and structural aspects of transcriptomic analysis [11]. Emerging techniques have made it possible to identify the dominant cell populations and spatial patterns of gene expression variations. However, the high cost of these techniques limits their use to large clinical cohorts. Integrating the strengths of scRNA-seq and ST, along with valuable clinical information from traditional bulk transcriptomics studies, holds great promise for providing a comprehensive transcriptional landscape of HCC.

To provide a unified portal for studying gene expression variations in HCC with both large population and single-cell resolution, we released HCCDB v2.0, an updated version containing 5573 bulk transcriptomic samples, 182,832 cells, and 69,352 spatial spots. In addition to the previous 4-dimension (4D) metric for bulk data, a single-cell level and 2-dimension (sc-2D) metric for single-cell data was also proposed for depicting gene expression variations across different cell types as well as the differential expression between tumor and normal hepatocytes. Based on this resource, we successfully identified cell subpopulations and tumor microenvironments associated with poor prognosis by integrating the three-way transcriptomic data. To facilitate the usage of this resource, a new searchable web portal was designed to visualize a set of pre-calculated results.

## Database content and computation methods

### The archived bulk expression datasets

For the release of HCCDB v2.0, we collected publicly accessible expression datasets of HCC up to March 2022. In total, 5573 bulk transcriptomic samples from 26 HCC datasets were archived (Table S1). The preprocessing procedures were described in our previous study [6]. Log_2_ transformation was applied to probe values from microarray and normalized read counts from RNA sequencing (RNA-seq), respectively. Clinical information, such as tumor stages and survival time, was also collected if available. We standardized the terminology of clinical information to enable across-dataset analysis. For instance, the unit of all survival time information was converted to month.

### Search and processing of scRNA-seq datasets

The scRNA-seq datasets of both human normal liver and HCC tissue were retrieved from the Gene Expression Omnibus (GEO) database. By exclusively considering datasets with 3′-end sequencing from the 10X Genomics platform, two scRNA-seq datasets were archived, including a normal liver dataset (HCCDB-SC1) [12] and an HCC dataset (HCCDB-SC2) [13]. To enrich the datasets in the database, we enrolled seven HCC patients and obtained seven tumor samples and three tumor-adjacent liver tissues (HCCDB-SC3, in-house data). The information on single-cell transcriptomic datasets was shown in Table S2. We carried out cell-level quality control by filtering mitochondrial messenger RNA (mt-mRNA) and unique feature counts (fc) [14]. Cells with elevated mt-mRNA levels (mt > 20) were excluded, as well as those with excessively high or low unique feature counts (fc < 200 or fc > 10,000). Principal component analysis (PCA) was carried out to project the spots onto a low-dimensional space defined by the first 30 principal components (PCs). We eliminated batch effects between datasets through the canonical-correlation analysis (CCA) algorithm. The clustering was performed using Seurat (v4.3.0) [15]. Two dimensionality reduction methods, Uniform Manifold Approximation and Projection (UMAP) and *t*-Distributed Stochastic Neighbor Embedding (*t*-SNE), were used to visualize the clustering. We manually annotated the cell clusters and identified 7 major lineages and 18 minor lineages in total.

### Processing of ST datasets

The ST data were collected from our prior study [16]. For ST, we released 17 tissue sections with a total of 69,352 tissue spots from 5 patients (HCC-1 to HCC-5). The detailed processing method was described as before. In total, four nontumor sections, four leading-edge sections, four tumor sections, one portal vein tumor thrombus section, and four sections from an intact tumor nodule were obtained (Table S3).

### Identification of driver factors regulating gene expression

DNA methylation and somatic copy number variation (CNV) are both considered as major factors affecting gene expression. To determine genetic factors that contribute to the regulation of transcript expression, we applied a multivariate linear regression model to DNA methylation and CNV data from The Cancer Genome Atlas (TCGA) datasets. Considering gene *G_i_*, the CpG methylation levels in the promoter region (*M*), the CNV (*C*), and the random error (*ω*), the equation was characterized as follows:

(1) $$G_{i}=C_{i}\;+\;M_{i}\;+\;\omega_{i}$$

Individual genetic factors with an adjusted *P* value < 0.05 (Benjamini–Hochberg correction) were considered as driver regulation factors.

### Identification of consistently differentially expressed genes

In HCCDB v2.0, the number of datasets with both tumor and adjacent samples was increased to 19. We used the *t*-test function in R for each dataset to determine if there was a significant difference in gene expression between the tumor and adjacent samples, followed by Benjamini–Hochberg correction. Consistently differentially expressed genes (cDEGs) were defined as those exhibiting expression measurements in eight datasets, with significant differential expression (adjusted *P* < 0.001 and |log_2_ fold change (FC)| > 0.6) in at least half of the datasets.

### Prognostic analysis

Six datasets (HCCDB6, HCCDB15, HCCDB18, HCCDB19, HCCDB24, and HCCDB25) with overall survival time information were used to evaluate the prognostic performance of each gene. The median expression value in each dataset was employed to classify high and low expression groups. The significance was determined by the log-rank test. Prognostic genes were defined as genes with adjusted *P* < 0.001 (Benjamini–Hochberg correction) in one dataset or adjusted *P* < 0.01 in more than two datasets. Genes with negative Cox coefficients were labeled as “favorable genes”, indicating a higher expression value correlated with a lower risk, while genes with positive Cox coefficients were labeled as “unfavorable genes”.

### Definition of 4D metric, sc-2D metric, and ST deregulation metric

In the previous release, we introduced a 4D metric for each gene, a potent tool to evaluate gene variation and summarize the expression pattern at the bulk level, including liver-specific metric, deregulation metric, tumor-specific metric, and HCC-specific metric. Because of the increased number of HCCDB datasets, the deregulation metric was revised. The definition of 4D metric was characterized as previously [6]. In addition, we proposed a sc-2D metric in single-cell scales, which conducted the two-dimensional metric for each gene in each cell type to decipher gene expression variance. Given the limited adjacent samples, cells from both adjacent and normal tissues were considered as the control group of HCC samples. The definitions for the two metrics are as follows.

Cell-specific metric quantifies the specificity of gene *i* in each cell type *j* in comparison with other cell types:

(2) $${\mathrm{Cell}-\mathrm{specific}\;\mathrm{metric}}_{j}=\log_{2}{\left(\frac{{\bar{x}}_{\;\mathrm{cell}\;j}}{{\bar{x}}_{\;\mathrm{other}\;\mathrm{cell}}}\right)}_{\mathrm{HCC}+\mathrm{adjacent}+\mathrm{normal}}$$

HCC deregulation metric quantifies the log_2_ FC of gene *i* in tumor tissues compared with normal and adjacent tissues in each cell type *j*:

(3) $${\mathrm{HCC}\;\mathrm{deregulation}\;\mathrm{metric}}_{j}=\log_{2}{\left(\frac{{\bar{x}}_{\;\mathrm{HCC}}}{{\bar{x}}_{\;\mathrm{adjacent}+\mathrm{normal}}}\right)}_{\mathrm{cell}\;j}$$

For ST sub-atlas, ST deregulation metric quantifies the log_2_ FC of gene *i* in tumor spots compared with adjacent spots in ST samples (HCC-1 to HCC-5), and is calculated as below:

(4) $$\mathrm{ST}\;\mathrm{deregulation}\;\mathrm{metric}\;=\;\bar{\log_{2}{\left(\frac{{\bar{x}}_{\;\mathrm{tumor}\;\mathrm{spots}}}{{\bar{x}}_{\;\mathrm{adjacent}\;\mathrm{spots}}}\right)}_{\mathrm{HCC}\;1–5}}$$

### Calculation of the highly regional gene score

We used the highly regional gene (HRG) algorithm to evaluate the regional distribution extent of individual genes [17]. The scoring function for gene g is defined as:

(5) $${\mathrm{Score}}_{g}=\sum_{i}\sum_{j\neq i}e_{gi}e_{gj}\;\times \;\mathrm{similarity}\left(c_{i},c_{j}\right)$$

where $e_{gi}$ and $e_{gj}$ are scaled gene expression levels of gene g in cells $c_{i}$ and $c_{j}$, respectively, and $\mathrm{similarity}\left(c_{i},c_{j}\right)$ is the spatial distance between cells $c_{i}$ and $c_{j}$. The value of $s\mathrm{imilarity}\left(c_{i},c_{j}\right)$ is 1 if cells $c_{i}\;$and $c_{j}$ are connected in space, and 0 otherwise. The expression $e_{gi}$ is positive if it is higher than the average gene expression, and negative otherwise. If the expression levels of gene g in cells $c_{i}$ and $c_{j}$ are both higher or lower than the average expression level, the product of $e_{gi}e_{gj}\;\times \;\mathrm{similarity}\left(c_{i},c_{j}\right)$ positively contributes to its score. If $c_{i}$ and $c_{j}$ happen to be similar, the contribution is greater. On the contrary, if the expression levels of gene g in $c_{i}$ and $c_{j}$ are either higher or lower than the average expression level, $e_{gi}e_{gj}\;\times \;\mathrm{similarity}\left(c_{i},c_{j}\right)$ exerts negative contribution to its score. Intuitively, a high score indicates that expression levels in two similar cells are similar, suggesting regional distribution patterns of genes.

The open-source HighlyRegionalGenes R package can be accessed on GitHub (https://github.com/JulieBaker1/HighlyRegionalGenes). We conducted 10 rounds of iteration and selected the top 2000 genes per round. The HRG score was log_2_-transformed and scaled accordingly.

### Identification of phenotype-related cells by Scissor

We applied Scissor to identify cell populations related to overall survival. Scissor is formulated as the following network regularized sparse regression model:

(6) $$\underset{\beta }{\min}-\frac{1}{n}l\left(\beta \right)+\lambda \left\{\alpha |\beta {|}_{1}+\frac{1-\alpha }{2}\beta^{T}L\beta \right\}$$

where *L* is a symmetric normalized Laplacian matrix defined as:

(7) $$L=D^{-\frac{1}{2}}\left(D-A\right)D^{-\frac{1}{2}}=I-D^{-\frac{1}{2}}AD^{-\frac{1}{2}}$$

Here, $A={\left(a_{ij}\right)}_{m\times m}$ is a binary or weighted adjacency matrix of a cell–cell similarity network G. The value of $a_{ij}$ equals 1 or a value ranging from 0 to 1 if cells i and j are connected in *G*, and $a_{ij}$ = 0 otherwise. The $D={\left(d_{ij}\right)}_{m\times m}$ is the degree matrix of G, where $d_{ii}=\sum_{j=1}^{m}a_{ij}$, and $d_{ij}=0$ for i≠j. The tuning parameter λ controls the overall strength of the penalty, and α balances the amount of regularization for smoothness and sparsity. Here, n is the number of bulk samples, while β denotes a vector of coefficients on cells and $l\left(\beta \right)$ denotes an appropriately chosen log-likelihood function.

For the analysis of clinical survival data, Cox regression was employed to determine the most phenotype-associated cell subpopulations from the single-cell data.

(8) $$l\left(\beta \right)=\sum_{i=1}^{n}\delta_{i}\left[\beta^{T}S_{i}-\log\left(\sum_{k\in R_{i}}\exp\left(\beta^{T}S_{k}\right)\right)\right]$$

where $\delta_{i}$ is the event indicator, with $S_{i}={\left(s_{i1},s_{i2},\ldots ,s_{im}\right)}^{T}$ as the correlation coefficients for sample i across all m cells. $R_{i}=k:\hat{T_{k}}\geq \hat{T_{i}}\;$denotes the risk set at time $\hat{T_{i}}$.

The non-zero coefficients of β solved by the aforementioned optimization model were used to select the cell subpopulations associated with the overall survival. Scissor^+^ (positive sign of β) cells are associated with poor survival, and Scissor^−^ (minus sign of β) cells are associated with good survival. A reliability significance test was further designed to control false associations. The final single-cell overall survival status was merged with the survival status calculated from all seven sets of bulk datasets.

The parameter α, which balances the impact of the l1 norms and network-based penalties, was set to 0.05. The cutoff value for the percentage of Scissor-selected cells among all cells was set to 0.2.

### Estimation of cell abundance of tumor microenvironment

To quantify the abundance of stromal and immune cells of data in HCCDB, we applied xCell (R package, v1.1.0) on the normalized expression data of each dataset to estimate scores for 38 infiltrating cell subtypes [18]. Kaplan–Meier analysis and log-rank test (R survival package, v3.4-0) were applied to assess the clinical relevance of CD8^+^ T cells.

## Implementation and results

### Overview of updated HCCDB database

**Figure 1:**
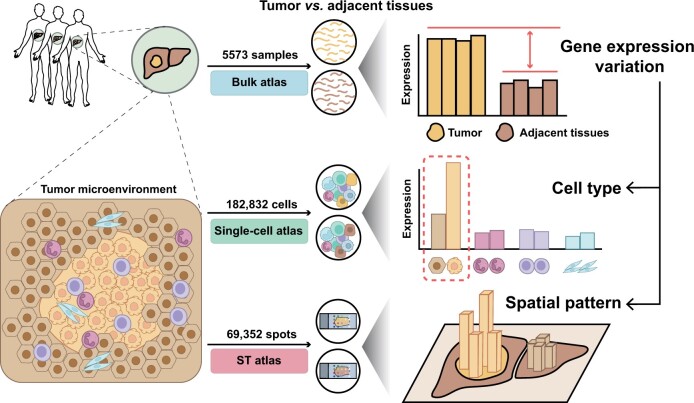
Overall design of HCCDB v2.0 HCCDB v2.0 offers transcriptomic data for both primary tumor tissues and adjacent liver tissues. It features three sub-atlases to give a comprehensive view of the transcriptomic landscape. The gene expression variations obtained through bulk transcriptomics can be further analyzed at the single-cell level through single-cell transcriptomics, providing a deeper understanding of the underlying mechanisms. ST, on the other hand, sheds light on the regional expression pattern and spatial architecture. ST, spatial transcriptomics.

The database has undergone both horizontal and longitudinal expansion. Horizontally, new bulk transcriptomic, scRNA-seq, and ST datasets have been added. Longitudinally, a new analysis pipeline has been implemented to decipher bulk gene expression variations using scRNA-seq and ST, providing a cellular-level resolution of gene expression patterns (**Figure 1**).

HCCDB v2.0 includes three sub-atlases: bulk transcriptomics, single-cell transcriptomics, and ST, all accessible from the HOME page. For queried genes, two search modes are available: single-gene search and multi-gene search. The result page for the original single gene search has been relocated to the bulk sub-atlas. The bulk sub-atlas includes summary information, expression patterns, and survival analysis, while the co-expression panel has been removed to streamline content. Moreover, third-party links and PubMed database links have been reviewed and updated. The single-cell sub-atlas presents summary expression patterns using UMAP, violin plots, and dot plots. In the ST sub-atlas, emphasis is placed on highlighting the advantages of ST in exploring tumor spatial heterogeneity. Hematoxylin and eosin (H&E) stain sections and point-to-point spatial gene expression distributions are provided. A parallel framework for the three sub-atlases allows users to switch between sub-atlases at every step of browsing, searching, and downloading.

### Assessment of meta-analysis stability for bulk transcriptomic datasets after update

Following cleaning procedures, 1656 samples from 11 datasets were involved, significantly increasing the database content compared to the previous version, which contained 3917 samples from 15 datasets. The new datasets included HCCDB19, HCCDB25, and HCCDB30 derived from RNA-seq platforms, with the remaining datasets using microarrays. Seven of the eleven datasets contained both adjacent and HCC samples with standardized clinical information. The expanded datasets introduced novel clinical phenotypes, including disease-free survival, tumor purity, and sorafenib response. In total, HCCDB v2.0 released 26 datasets with 5573 samples and 16 clinical phenotypes, 19 of which comprised both adjacent and HCC samples (Tables S1–S3). In addition, we attached a tag about genomic factors controlling gene expression to the result page of bulk transcriptomics. For example, the up-regulation of *GPC3* in HCC is putatively driven by a methylation event in the promoter region.

**Figure 2:**
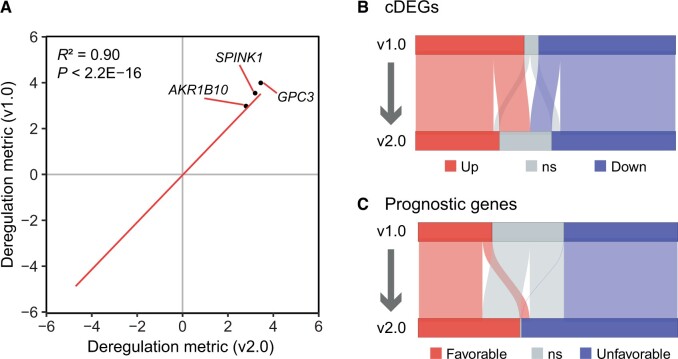
Analysis of bulk transcriptomic results after the expansion of datasets **A**. Scatter plot depicting the deregulation metrics between two versions (v1.0 for Y-axis and v2.0 for X-axis). The linear regression line is shown in red. The top 3 genes with the highest deregulation metric in two versions are pinpointed. Statistical significance was assessed using Pearson correlation (*P* < 2.2E−16). **B**. and **C**. The alluvial plots showing changes in cDEGs (B) and prognostic genes (C) after the revision. ns, not significant; cDEG, consistently differentially expressed gene.

We proposed metrics to classify the gene expression pattern of HCC, including the 4D metric, cDEGs, and prognostic genes, by integrating multiple datasets. We evaluated the impact of expanding the dataset size by 42% (1656/3917) on these three metrics. The revised deregulation metric showed a strong correlation with the original one (Pearson correlation test, *R*^2^ = 0.90, *P* < 2.2 E−16) (Table S4). The top three up-regulated genes, *GPC3*, *SPINK1*, and *AKR1B10*, remained the most significantly up-regulated after the revision (**Figure 2**A). The number of cDEGs decreased from 1259 to 1065, with some changes in gene identity (Figure 2B), but no up-regulated genes were converted to down-regulated, and *vice versa*, showing the relative stability of cDEG identity. The number of prognostic genes increased from 1346 to 1893, indicating the discovery of more prognostic genes with the expansion of datasets (Figure 2C). In summary, our results demonstrate that the gene expression pattern is essentially stable after expanding the datasets, with additional information and robustness.

### Decipher the gene expression variations at single-cell level

To gain a deeper understanding of HCC’s transcriptional landscape, we added a single-cell atlas featuring 182,832 cells from three scRNA-seq datasets: HCCDB-SC1 (normal liver data) [12], HCCDB-SC2 (HCC data) [13], and HCCDB-SC3 (HCC and adjacent tissues, in-house data). The analysis identified 9 major and 19 minor cell lineages. We enhanced the scRNA-seq result page with a graphical interface, allowing users to access selective information such as dataset, tissue, patient ID, Seurat cluster, and cell type. For each queried gene, four graphs displaying gene expression abundance are provided: a UMAP plot, a *t*-SNE plot, a violin plot, and a dot plot. These plots can be easily downloaded as high-quality graphics.

**Figure 3:**
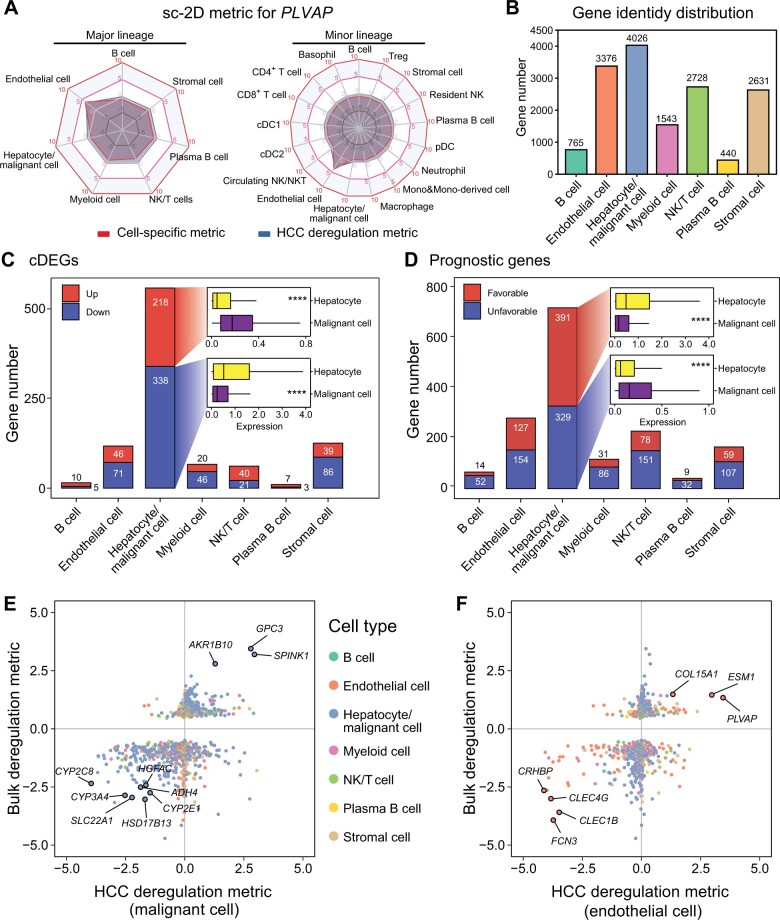
Analysis of sc-2D metric to decompose bulk gene expression patterns **A**. Radar plots illustrating the sc-2D pattern of an endothelial-specific gene, *PLVAP*, for the major lineage (left) and the minor panel (right). **B**. Distribution of gene identity. The cell type with the highest cell-specific metric is determined as cell-specific for individual genes. **C**. and **D**. Distribution of gene identity for cDEGs (C) and prognostic genes (D). The box charts inside show the comparison of the average expression of cDEGs (C) and prognostic genes (D) in hepatocytes in normal liver tissues and in malignant cells in tumor tissues. *P* value was calculated by the two-tailed Wilcoxon rank sum test. ****, *P* < 0.0001. **E**. and **F**. Scatter plots depicting the relationship between the deregulation metric derived from bulk transcriptomics and the HCC deregulation metric of malignant cells (E) and endothelial cells (F). sc-2D, single-cell level and 2-dimension; HCC, hepatocellular carcinoma; NK, natural killer; DC, dendritic cell.

To measure the extent of tumor microenvironment deregulation in HCC, we developed the sc-2D metric, including the cell-specific metric and HCC deregulation metric, quantifying the cell specificity and cellular deregulation degree for individual genes (Tables S5 and S6). As an example, the *PLVAP* gene, which is expressed by tumor endothelium [19,20], exhibited a notably high cell-specific metric and HCC deregulation metric in both the major and minor cell lineages (**Figure 3**A). Each gene was assigned a unique identity tag, with the cell type exhibiting the highest cell-specific metric referred to as the “master cell type”. Most of the genes (82.28%, 12,761/15,509) were primarily expressed in hepatocytes, malignant cells, endothelial cells, stromal cells, and natural killer (NK)/T cells (Figure 3B), demonstrating that these cell types mainly contributed to the bulk variations of HCC. Additionally, when focusing on cDEGs and prognostic genes from bulk transcriptomics, we found that 58.5% (556/950) of cDEGs and 44.4% (720/1620) of prognostic genes were primarily expressed in hepatocytes or malignant cells, and exhibited significant differences in expression between normal and tumor cells, highlighting the crucial role of malignant cells in tumor dysregulation and prognosis (Figure 3C and D).

The deregulation degree of HCC was quantified by the deregulation metric derived from bulk transcriptomics. However, the contribution of each cell type to the deregulation of HCC remained unclear. Here, we deciphered the bulk deregulation metric by HCC deregulation metric of each cell type. The genes *GPC3* and *SPINK1* were mainly expressed in malignant cells, exhibiting both high bulk deregulation metric and HCC deregulation metric of malignant cells (Figure 3E). Distinctive expression patterns of genes were shown in other cell types, such as up-regulated *THY1* in stromal cells and up-regulated *SPP1* in myeloid cells (Figure S1A–E). Intriguingly, several genes, including *CLEG4G* and *FCN3*, were expressed in normal endothelial cells but down-regulated in tumor tissues (Figure 3F). Notably, *FCN3* has been identified as a tumor-suppressive gene in lung adenocarcinoma and is associated with a favorable prognosis in HCCDB [21]. Our results suggest that endothelial-specific genes, such as *FCN3*, may play important roles in preventing tumor progression. However, the roles of endothelial cells and these endothelial-specific genes in HCC remain largely unexplored. Overall, the expansion of single-cell atlas allows decomposing the bulk variations to the cellular level and identifying potential therapeutic targets.

### ST benefits the investigation of tumor heterogeneity

**Figure 4:**
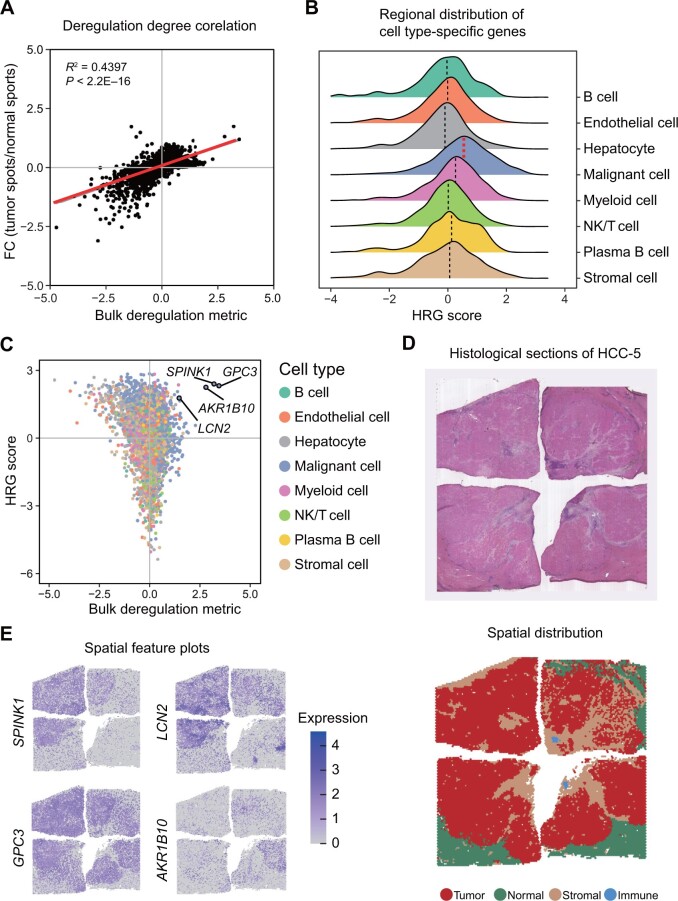
ST atlas exhibits intra-tumor heterogeneity **A**. Scatter plot depicting the correlation between the deregulation metric derived from bulk transcriptomics (X-axis) and the ST deregulation metric derived from ST (Y-axis). The linear regression line is represented in red. Pearson correlation was used to assess statistical significance (*P* < 2.2E−16). **B**. Regional distribution of cell type-specific genes quantified by HRG score. The median value for each group is indicated by a dashed line. Malignant cell-specific genes show the highest median HRG score (colored in red). **C**. Scatter plot depicting the relationship between the deregulation metric derived from bulk transcriptomics (X-axis) and the HRG score (Y-axis). Four genes with both high bulk deregulation values and HRG scores were pinpointed. **D**. H&E staining (top) and the spatial cluster distribution (bottom) of HCC-5. Four tissue spot types were identified including tumor, normal, stromal, and immune spots. **E**. Spatial gene expression distribution of four genes (*GPC3*, *SPINK1*, *AKR1B10*, and *LCN2*) on HCC-5 (related to D). FC, fold change; HRG, highly regional gene; H&E, hematoxylin and eosin.

For ST atlas, we collected 69,352 tissue spots and 17 tissue histological sections from 5 patients (HCC-1 to HCC-5), with annotations for immune, tumor, adjacent, and stromal regions. The H&E stain sections and spatial cluster distribution plots for each patient are presented in the ST atlas, along with interactive UMAP and *t*-SNE plots. To visualize gene expression in different patients and tissue regions, we provided spatial feature plots, dot plots, and violin plots. The comparison of ST deregulation metric with the bulk deregulation metric revealed a high concordance between ST and bulk transcriptomics (**Figure 4**A).

To assess the ability of ST to detect intra-tumor heterogeneity, we utilized the HRG algorithm to analyze a 1-cm-diameter tumor nodule from patient HCC-5 [17]. The HRG algorithm, which identifies regionally distributed genes by constructing a cell–cell similarity graph, revealed that malignant cell-specific genes determined by scRNA-seq had the highest HRG score among all cell types (Figure 4B). Additionally, several dysregulated genes, such as *GPC3*, *AKR1B10*, *SPINK1*, and *LCN2*, showed both high HRG scores and bulk deregulation degrees (Figure 4C; Table S7). These genes also exhibited different spatial expression distributions in this tumor nodule (Figure 4D and E). Despite extensive up-regulation at the bulk level in different clinical stages, the spatial distribution patterns of these genes at early developmental stages can be diverse. In conclusion, the combination of ST and bulk transcriptomic data has improved our understanding of tumor heterogeneity and provided insights into gene expression variations.

### Identification of a population of prognosis-related cells by combining bulk transcriptomic and scRNA-seq datasets

**Figure 5:**
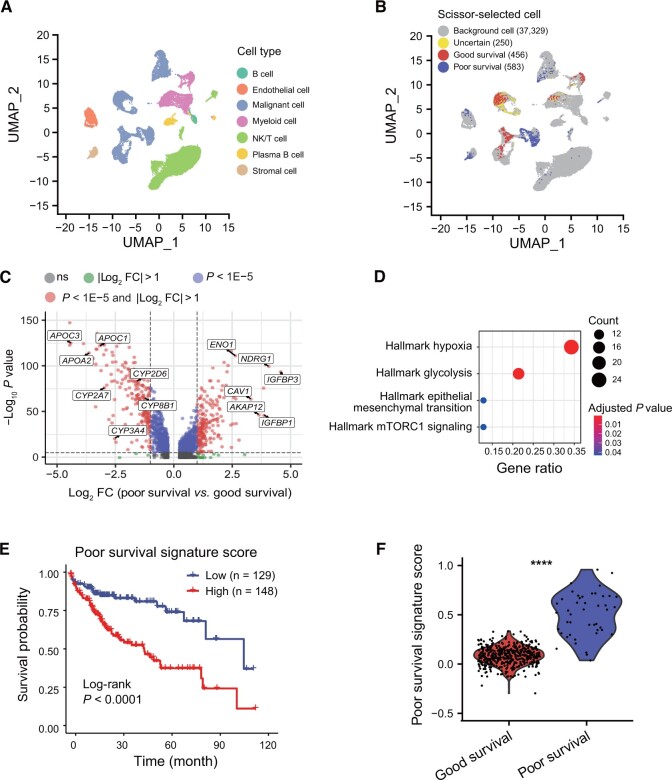
Identification of prognosis-related cells in malignant cells guided by HCCDB survival outcomes **A**. UMAP visualization of major cell types from HCCDB-SC2. **B**. UMAP visualization of the Scissor-selected cells. **C**. Volcano plot of differential gene expression in poor survival cells *vs.* good survival cells. **D**. Enrichment bar plot of poor survival cell-related up-regulated genes in hallmark pathways. **E**. Kaplan–Meier survival curves demonstrating the clinical relevance of the poor survival signature on the HCCDB15 dataset. **F**. The poor survival signature scores in HCCDB-SC3 Scissor-selected cells. The FDR was the adjusted *P* value calculated by the two-tailed Wilcoxon rank sum test. ****, *P* < 0.0001. UMAP, Uniform Manifold Approximation and Projection; FDR, false discovery rate.

HCCs are heterogeneous and include subpopulations such as cancer stem cells, which are known to drive tumor progression and poor prognosis [22]. To identify cells related to poor survival, we applied the Scissor method [23] to eight bulk datasets with survival information, leaving out HCCDB15 derived from TCGA as the validation dataset. To avoid batch effects among bulk datasets, we transferred the survival information of other bulk datasets to HCCDB-SC2 individually. Cells with contradicted transferred phenotype in different bulk datasets were labeled as “uncertain”, while those related to good survival or poor survival in more than one bulk dataset were designated as good survival or poor survival cells, respectively. The majority of the Scissor-selected cells were malignant epithelial cells (**Figure 5**A and B, Figure S2A), indicating the decisive influence of epithelial cells on patients’ survival. Besides, the phenotype of Scissor-selected cells within a patient was consistently related to either good survival or poor survival (Figure S2B).

To identify key factors associated with poor survival, we compared poor survival cells to good survival cells (Figure 5C; Table S8). Notably, we found relatively higher expression of hepatocyte function-related genes involved in glycogen/lipid/alcohol metabolism (APO/ALDH/ADH family genes) and detoxification (CYP family genes) in good survival cells, while multiple important hypoxia-related genes were highly expressed in poor survival cells (Figure S2C). Functional enrichment analysis also indicated that poor survival-related genes were enriched in the hypoxia pathway (Figure 5D). To validate the poor survival gene signature, we scored patients in the validation dataset HCCDB15 and found that patients with higher scores had significantly poor survival (Figure 5E). Meanwhile, using the same procedure for choosing good survival and poor survival cells in the HCCDB-SC3 dataset, we found that poor survival cells had a significantly higher score of the poor survival gene signature derived from the HCCDB-SC2 dataset (Figure 5F), indicating the robustness of the poor survival signature. Taken together, the integration of scRNA-seq datasets and bulk datasets identified a specific population of prognosis-related cells and provided a poor survival signature for prognostic prediction.

### Tumor microenvironment programs related to prognosis

**Figure 6:**
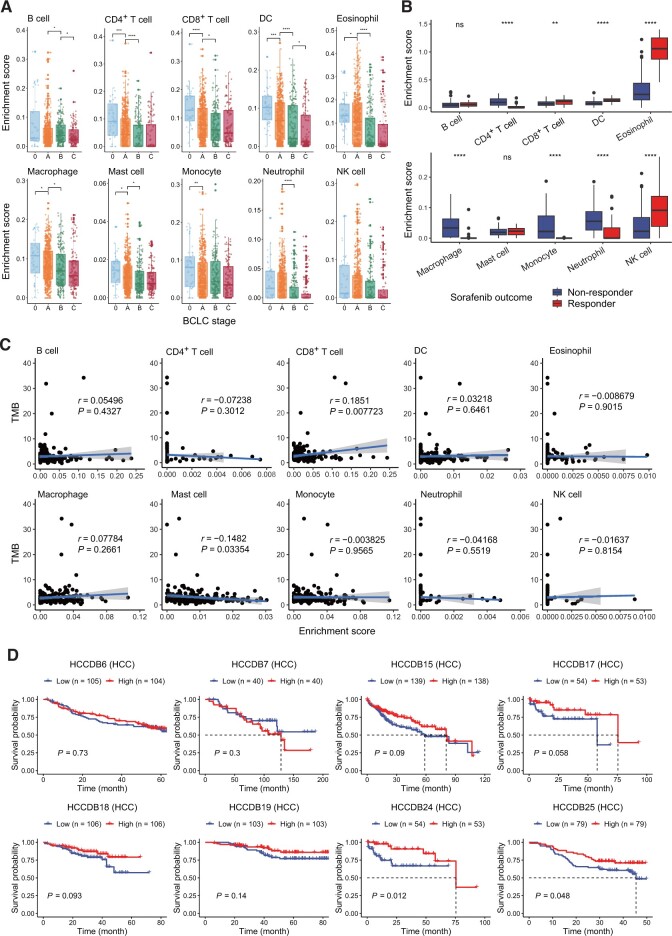
Deconvolution of HCC immune microenvironment **A**. Boxplots showing the connectivity between 10 tumor-infiltrating immune cells and the BCLC stage. The *P* value between the two groups was calculated with Wilcoxon tests. *, *P* < 0.05; **, *P* < 0.01; ***, *P* < 0.001; ****, *P* < 0.0001. **B**. Boxplots showing the differences in the abundances of 10 immune cells between the sorafenib response group and the non-response group. The *P* value between the two groups was calculated with Wilcoxon tests. **, *P* < 0.01; ****, *P* < 0.0001. **C**. Scatter plots illustrating the relationships between the abundances of 10 tumor-infiltrating immune cells and TMB. The shaded bands represent 95% confidence intervals of linear regression slopes. *P* values were from *t*-tests. **D**. Kaplan–Meier plots showing the relationships between the abundances of CD8^+^ T cells and the clinical outcomes in HCC patients, with the data divided by the median abundance of CD8^+^ T cells. The statistical significance was determined by log-rank tests. BCLC, Barcelona Clinic Liver Cancer; TMB, tumor mutational burden.

Solid tumors are commonly infiltrated by immune cells, including T and B lymphocytes, NK cells, dendritic cells (DCs), macrophages, neutrophils, eosinophils, and mast cells [24]. To assess the relationship between tumor-infiltrating immune cells and tumor progression, we applied xCell to eight HCCDB datasets with phenotypic information to calculate the enrichment scores of 38 types of immune cells and stromal cells (Table S9). The Barcelona Clinic Liver Cancer (BCLC) system offers a prognostic stratification of HCC patients [25]. We observed that the enrichment scores of most immune cells decreased significantly with the deterioration of the disease in the four datasets with BCLC stage information (**Figure 6**A), indicating a decrease in the abundance of immune infiltration with the deterioration of HCC. In the HCCDB28 dataset, we observed that the abundances of CD8^+^ T cells, DCs, eosinophils, and NK cells in the samples that responded to sorafenib were significantly higher than those in the non-responsive samples. On the other hand, the abundances of other immune cells, such as CD4^+^ T cells, macrophages, monocytes, and neutrophils, were lower in the responder group (Figure 6B). Tumor mutational burden (TMB) is an important biomarker for response to PD-1/PD-L1 inhibitors [26]. We also explored the correlation between TMB and tumor-infiltrating immune cells using the HCCDB19 dataset. The enrichment score of CD8^+^ T cells was significantly positively associated with TMB, whereas that of mast cells was negatively correlated (Figure 6C). Meanwhile, we found that the enrichment of CD8^+^ T cells was significantly associated with good survival in survival analyses (Figure 6D). Taken together, the integration of multiple datasets reveals that the tumor-infiltrating immune cells are closely related to clinical phenotype and tumor progression in HCC.

## Perspectives and concluding remarks

Large-scale transcriptomic data have greatly facilitated the analysis and discovery of new therapeutic targets and biomarkers. Particularly, the rapid growth of bulk transcriptomic data provides a wealth of clinical information, making it advantageous for the integrative analysis of transcriptome and clinical information. However, the limited resolution of bulk transcriptomics has hindered further analysis in light of the growing need to understand tumor heterogeneity and immune microenvironment. Therefore, we released HCCDB v2.0, a comprehensive transcriptomic database with 5573 bulk transcriptomic samples, 182,832 cells, and 69,352 spatial spots. To our knowledge, this is the first oncology database combining scRNA-seq and ST with bulk transcriptomics to describe transcriptional landscapes. To fully grasp the gene expression pattern, our parallel framework allows users to easily switch between different omics. Users can browse the comprehensive expression profiles of individual genes, including gene information and clinical information from the bulk transcriptomics atlas, cell annotation information from the scRNA-seq atlas, and spatial distribution from the ST atlas. Combined with genomic information and third-party links, users can achieve a seamless, one-stop searching workflow in HCCDB v2.0. The 4D metric and sc-2D metric provide a new perspective for the integration analysis of different transcriptomic data, which is a bold and innovative attempt.

Here, we present two cases for potential data exploration using the HCCDB v2.0 database. Scissor provides a good analytical model, enabling the extensive utilization of a large amount of clinical information from HCCDB v2.0 and transcriptomic data of scRNA-seq. By leveraging the abundant phenotypes of the bulk datasets and the precision of single-cell datasets, we successfully identified phenotype-related cells. Scissor can transfer the phenotype to single cells, as demonstrated by the successful transfer of survival information in bulk datasets to HCCDB-SC2 scRNA-seq datasets, leading to the identification of epithelial cells related with poor survival, with up-regulated genes enriched in hypoxia hallmarks. These cells could serve as potential drug targets for further investigation. The poor survival signature can be successfully transferred to independent bulk datasets and scRNA-seq datasets, indicating the robustness of the signature. Furthermore, xCell can identify the enrichment of multiple cell types in bulk data, allowing for the association of the clinical information in HCCDB v2.0 with one or several cell types. We observed lower immune infiltration correlated with elevated BCLC stage. In the HCCDB28 dataset, the abundances of different immune cells showed different distributions in sorafenib and control groups. Our data also indicated that CD8^+^ T cell enrichment significantly predicted high TMB and a good prognosis in HCC patients. As more phenotypes, such as response to anti-PD1 therapy, are collected in the future, we can identify cells related to the outcome of anti-PD1 therapy.

HCCDB v2.0 has demonstrated the viability of data exploration to offer resources and convenience for addressing pressing scientific issues. In the future, our group will keep the content updated, add new transcriptomic data, and integrate other omics data to develop new integrated analysis methodologies. Subsequent iterations of the HCCDB database may indeed demonstrate even greater potential.

## Supplementary Material

qzae011_Supplementary_Data

## Data Availability

HCCDB v2.0 is available at http://lifeome.net/database/hccdb2.

## CRediT author statement

**Ziming Jiang:** Conceptualization, Methodology, Software, Formal analysis, Investigation, Data curation, Writing – original draft, Visualization. **Yanhong Wu:** Formal analysis, Investigation, Data curation, Writing – original draft, Visualization. **Yuxin Miao:** Formal analysis, Writing – original draft, Visualization. **Kaige Deng:** Investigation. **Fan Yang:** Investigation. **Shuhuan Xu:** Software. **Yupeng Wang:** Software. **Renke You:** Software. **Lei Zhang:** Software. **Yuhan Fan:** Investigation, Data curation. **Wenbo Guo:** Methodology, Data curation. **Qiuyu Lian:** Conceptualization, Methodology, Data curation. **Lei Chen:** Conceptualization, Writing – review & editing. **Xuegong Zhang:** Conceptualization, Writing – review & editing. **Yongchang Zheng:** Conceptualization, Resources, Writing – review & editing, Funding acquisition. **Jin Gu:** Conceptualization, Methodology, Writing – review & editing, Supervision, Project administration, Funding acquisition. All authors have read and approved the final manuscript.

## ORCID

0000-0003-4762-7516 (Ziming Jiang)

0000-0003-1677-0467 (Yanhong Wu)

0000-0003-1738-2466 (Yuxin Miao)

0000-0003-4461-1746 (Kaige Deng)

0000-0002-7467-0117 (Fan Yang)

0009-0006-2519-3760 (Shuhuan Xu)

0009-0009-5312-4334 (Yupeng Wang)

0009-0006-6795-8864 (Renke You)

0009-0005-9921-0810 (Lei Zhang)

0009-0000-8290-8896 (Yuhan Fan)

0000-0002-7973-6795 (Wenbo Guo)

0000-0002-5279-1989 (Qiuyu Lian)

0000-0002-9380-9559 (Lei Chen)

0000-0002-9684-5643 (Xuegong Zhang)

0000-0002-5916-2392 (Yongchang Zheng)

0000-0003-3968-8036 (Jin Gu)

## References

[1] Bray F , Ferlay J, Soerjomataram I, Siegel RL, Torre LA, Jemal A. Global cancer statistics 2018: GLOBOCAN estimates of incidence and mortality worldwide for 36 cancers in 185 countries. CA Cancer J Clin 2018;68:394–424. 30207593 10.3322/caac.21492

[2] Rebouissou S , Nault JC. Advances in molecular classification and precision oncology in hepatocellular carcinoma. J Hepatol 2020;72:215–29. 31954487 10.1016/j.jhep.2019.08.017

[3] Sia D , Jiao Y, Martinez-Quetglas I, Kuchuk O, Villacorta-Martin C, Castro de Moura M, et al Identification of an immune-specific class of hepatocellular carcinoma, based on molecular features. Gastroenterology 2017;153:812–26. 28624577 10.1053/j.gastro.2017.06.007 PMC12166766

[4] Boyault S , Rickman DS, de Reyniès A, Balabaud C, Rebouissou S, Jeannot E, et al Transcriptome classification of HCC is related to gene alterations and to new therapeutic targets. Hepatology 2007;45:42–52. 17187432 10.1002/hep.21467

[5] Gurevitch J , Koricheva J, Nakagawa S, Stewart G. Meta-analysis and the science of research synthesis. Nature 2018;555:175–82. 29517004 10.1038/nature25753

[6] Lian Q , Wang S, Zhang G, Wang D, Luo G, Tang J, et al HCCDB: a database of hepatocellular carcinoma expression atlas. Genomics Proteomics Bioinformatics 2018;16:269–75. 30266410 10.1016/j.gpb.2018.07.003 PMC6205074

[7] Lv H , Lv G, Chen C, Zong Q, Jiang G, Ye D, et al NAD^+^ metabolism maintains inducible PD-L1 expression to drive tumor immune evasion. Cell Metab 2021;33:110–27. 33171124 10.1016/j.cmet.2020.10.021

[8] Wu T , Luo G, Lian Q, Sui C, Tang J, Zhu Y, et al Discovery of a carbamoyl phosphate synthetase 1-deficient HCC subtype with therapeutic potential through integrative genomic and experimental analysis. Hepatology 2021;74:3249–68. 34343359 10.1002/hep.32088

[9] Stegle O , Teichmann SA, Marioni JC. Computational and analytical challenges in single-cell transcriptomics. Nat Rev Genet 2015;16:133–45. 25628217 10.1038/nrg3833

[10] Tang F , Barbacioru C, Wang Y, Nordman E, Lee C, Xu N, et al mRNA-seq whole-transcriptome analysis of a single cell. Nat Methods 2009;6:377–82. 19349980 10.1038/nmeth.1315

[11] Crosetto N , Bienko M, van Oudenaarden A. Spatially resolved transcriptomics and beyond. Nat Rev Genet 2015;16:57–66. 25446315 10.1038/nrg3832

[12] Guilliams M , Bonnardel J, Haest B, Vanderborght B, Wagner C, Remmerie A, et al Spatial proteogenomics reveals distinct and evolutionarily conserved hepatic macrophage niches. Cell 2022;185:379–96. 35021063 10.1016/j.cell.2021.12.018 PMC8809252

[13] Ma L , Wang L, Khatib SA, Chang CW, Heinrich S, Dominguez DA, et al Single-cell atlas of tumor cell evolution in response to therapy in hepatocellular carcinoma and intrahepatic cholangiocarcinoma. J Hepatol 2021;75:1397–408. 34216724 10.1016/j.jhep.2021.06.028 PMC8604764

[14] Guo W , Wang D, Wang S, Shan Y, Liu C, Gu J. scCancer: a package for automated processing of single-cell RNA-seq data in cancer. Brief Bioinform 2021;22:bbaa127. 34020534 10.1093/bib/bbaa127

[15] Hao Y , Hao S, Andersen-Nissen E, Mauck WM 3rd, Zheng S, Butler A, et al Integrated analysis of multimodal single-cell data. Cell 2021;184:3573–87. 34062119 10.1016/j.cell.2021.04.048 PMC8238499

[16] Wu R , Guo W, Qiu X, Wang S, Sui C, Lian Q, et al Comprehensive analysis of spatial architecture in primary liver cancer. Sci Adv 2021;7:eabg3750. 34919432 10.1126/sciadv.abg3750 PMC8683021

[17] Wu Y , Hu Q, Wang S, Liu C, Shan Y, Guo W, et al Highly Regional Genes: graph-based gene selection for single-cell RNA-seq data. J Genet Genomics 2022;49:891–9. 35144027 10.1016/j.jgg.2022.01.004

[18] Aran D , Hu Z, Butte AJ. xCell: digitally portraying the tissue cellular heterogeneity landscape. Genome Biol 2017;18:220. 29141660 10.1186/s13059-017-1349-1 PMC5688663

[19] Sharma A , Seow JJW, Dutertre CA, Pai R, Blériot C, Mishra A, et al Onco-fetal reprogramming of endothelial cells drives immunosuppressive macrophages in hepatocellular carcinoma. Cell 2020;183:377–94. 32976798 10.1016/j.cell.2020.08.040

[20] Strickland LA , Jubb AM, Hongo JA, Zhong F, Burwick J, Fu L, et al Plasmalemmal vesicle-associated protein (PLVAP) is expressed by tumour endothelium and is upregulated by vascular endothelial growth factor-A (VEGF). J Pathol 2005;206:466–75. 15971170 10.1002/path.1805

[21] Jang H , Jun Y, Kim S, Kim E, Jung Y, Park BJ, et al *FCN3* functions as a tumor suppressor of lung adenocarcinoma through induction of endoplasmic reticulum stress. Cell Death Dis 2021;12:407. 33859174 10.1038/s41419-021-03675-y PMC8050313

[22] Lee TK , Guan XY, Ma S. Cancer stem cells in hepatocellular carcinoma – from origin to clinical implications. Nat Rev Gastroenterol Hepatol 2022;19:26–44. 34504325 10.1038/s41575-021-00508-3

[23] Sun D , Guan X, Moran AE, Wu LY, Qian DZ, Schedin P, et al Identifying phenotype-associated subpopulations by integrating bulk and single-cell sequencing data. Nat Biotechnol 2022;40:527–38. 34764492 10.1038/s41587-021-01091-3 PMC9010342

[24] Pagès F , Galon J, Dieu-Nosjean MC, Tartour E, Sautès-Fridman C, Fridman WH. Immune infiltration in human tumors: a prognostic factor that should not be ignored. Oncogene 2010;29:1093–102. 19946335 10.1038/onc.2009.416

[25] Cillo U , Vitale A, Grigoletto F, Farinati F, Brolese A, Zanus G, et al Prospective validation of the Barcelona Clinic Liver Cancer staging system. J Hepatol 2006;44:723–31. 16488051 10.1016/j.jhep.2005.12.015

[26] Fumet JD , Truntzer C, Yarchoan M, Ghiringhelli F. Tumour mutational burden as a biomarker for immunotherapy: current data and emerging concepts. Eur J Cancer 2020;131:40–50. 32278982 10.1016/j.ejca.2020.02.038 PMC9473693

