# Supplementary material for: HCCDB v2.0: Decompose Expression Variations by Single-cell RNA-seq and Spatial Transcriptomics in HCC
Source: Genomics Proteomics Bioinformatics. 2024 Feb 3;22(1):qzae011. doi: 10.1093/gpbjnl/qzae011 (PMC11423853; doi:10.1093/gpbjnl/qzae011)
Supplement: qzae011_Supplementary_Data [file qzae011_supplementary_data.zip › Supplementary Captions.docx]

**Supplementary material**

**Figure S1**  **Analysis of sc-2D metric in different cell types**

The scatter plot depicts the relationship between the deregulation metric derived from bulk transcriptomics and the HCC deregulation metric of stromal cells (**A**), myeloid cells (**B**), NK/T cells (**C**), B cells (**D**), and plasma B cells (**E**).

**Figure S2**  **Distribution of Scissor-selected cells**

**A.** The constitution of Scissor-selected cells in different cell types. **B.** The constitution of Scissor-selected epithelial cells in different patients. **C.** Violin plots of expression levels of up-regulated genes in good survival cells and poor survival cells. OS, overall survival.

**Table S1 Summary of bulk transcriptomics datasets**

**Table S2 Summary of single-cell transcriptomics datasets**

**Table S3 Summary of spatial transcriptomics datasets**

**Table S4 The revised 4D metrics**

**Table S5 Cell-specific metric of major cell lineages**

**Table S6 HCC deregulation metric of major cell lineages**

**Table S7 Scaled HRG scores**

**Table S8 Poor survival markers**

**Table S9 Summary of tumor microenvironment information**
