# Supplementary material for: HCCDB v2.0: Decompose Expression Variations by Single-cell RNA-seq and Spatial Transcriptomics in HCC
Source: Genomics Proteomics Bioinformatics. 2024 Feb 3;22(1):qzae011. doi: 10.1093/gpbjnl/qzae011 (PMC11423853; doi:10.1093/gpbjnl/qzae011)
Supplement: qzae011_Supplementary_Data [file qzae011_supplementary_data.zip › Figure S1.pdf]

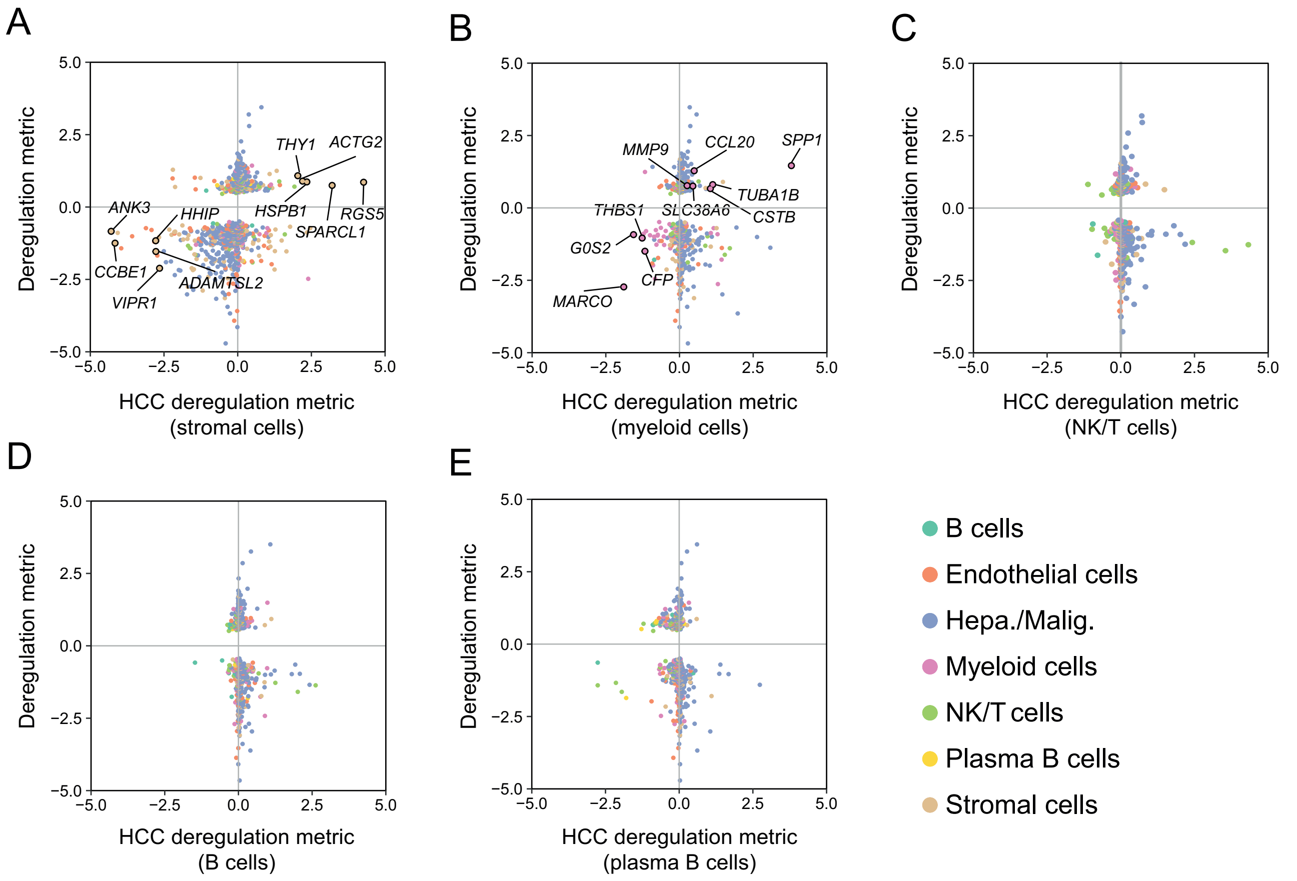
A
B
C
D
E
Deregulation metric
HCC deregulation metric (stromal cells)
HCC deregulation metric (myeloid cells)
HCC deregulation metric (NK/T cells)
HCC deregulation metric (B cells)
HCC deregulation metric (plasma B cells)
5.0
2.5
0.0
−2.5
−5.0
THY1
ACTG2
ANK3
HHIP
HSPB1
RGS5
SPARCL1
CCBE1
ADAMTSL2
VIPR1
MMP9
CCL20
SPP1
TUBA1B
THBS1
SLC38A6
CSTB
G0S2
CFP
MARCO
B cells
Endothelial cells
Hepa./Malig.
Myeloid cells
NK/T cells
Plasma B cells
Stromal cells
