# Supplementary figures and images for: HCCDB v2.0: Decompose Expression Variations by Single-cell RNA-seq and Spatial Transcriptomics in HCC
Source: Genomics Proteomics Bioinformatics. 2024 Feb 3;22(1):qzae011. doi: 10.1093/gpbjnl/qzae011 (PMC11423853; doi:10.1093/gpbjnl/qzae011)

A

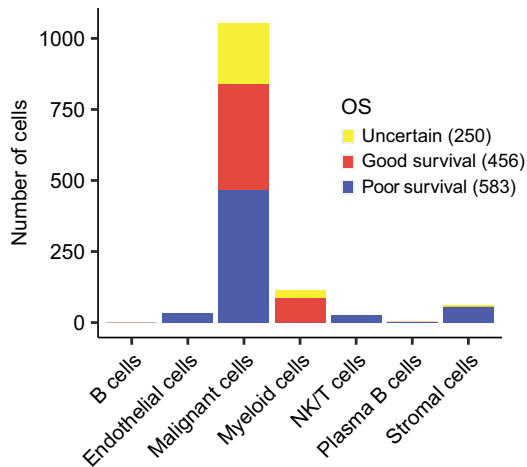

B

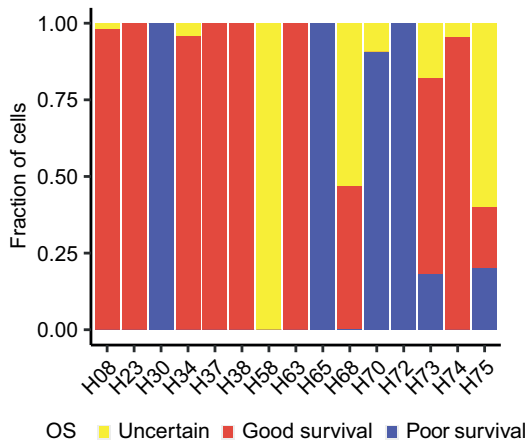

C

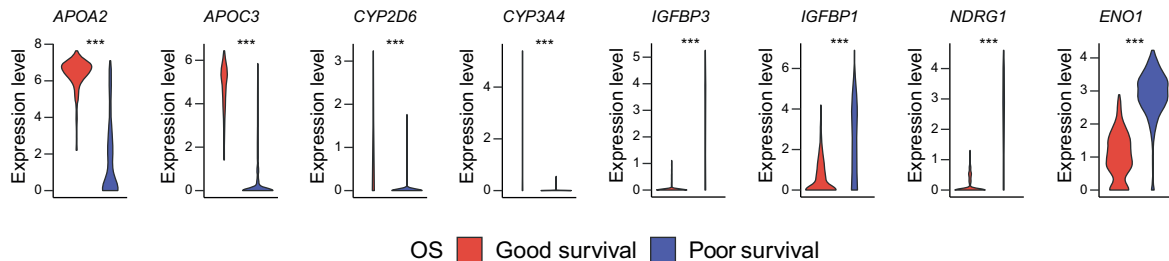

Supplement: qzae011_Supplementary_Data [file qzae011_supplementary_data.zip › Figure S2.pdf]
